# Environmental Governance, Public Health Expenditure, and Economic Growth: Analysis in an OLG Model

**DOI:** 10.3390/ijerph20043033

**Published:** 2023-02-09

**Authors:** Zhao Zhang, Caoyuan Ma, Aiping Wang

**Affiliations:** 1 School of Economics and Finance, Xi’an Jiaotong University, Xi’an 710061, China; 2 School of Economics, Beijing Technology and Business University, Beijing 100048, China

**Keywords:** environmental governance, health human capital, economic growth, overlapping generation model, numerical simulation

## Abstract

This article studies the relationship between environmental governance, public health expenditure, and economic growth by introducing human health status into a three-period overlapping generation dynamic general equilibrium (OLG-DGE) model and conducting a policy simulation analysis in a Chinese scenario. The main findings are generalized as follows: (i) The increase in pollution emissions per unit of output will not only lead to the deterioration of public health but also hinder long-term economic growth, while the efficiency of pollution control will improve health and output per labor unit; (ii) Although levying environmental tax will improve health status and life expectancy, it has a non-linear impact on pollution emissions and output per labor unit, which means that there are trade-offs among environmental governance, public health improvement, and economic output; and (iii) Although the increase in the proportion of public health expenditure will improve health status, its impact on life expectancy and economic output is affected by the level of environmental tax. Only when the environmental tax rate is relatively low, will increasing the proportion of public health expenditure extend life expectancy and output per labor unit.

## 1. Introduction

Environmental pollution is an important factor restricting global sustainable development. Numerous facts have proven that environmental pollution has been endangering human health in many ways. Therefore, environmental governance is becoming a consensus for most countries. Among the 17 Sustainable Development Goals (SDG) advocated by the United Nations [1], there are at least four that relate to environmental governance: good health and well-being (Goal 3), clean water and sanitation (Goal 6), affordable and clean energy (Goal 7), and climate action (Goal 13). Furthermore, Goal 8 (decent work and economic growth), Goal 9 (industry, innovation, and infrastructure), Goal 11(sustainable cities and communities), and Goal 12 (responsible consumption and production) also emphasize the effective use of resources and the sustainable development of the environment.

**Figure 1:**
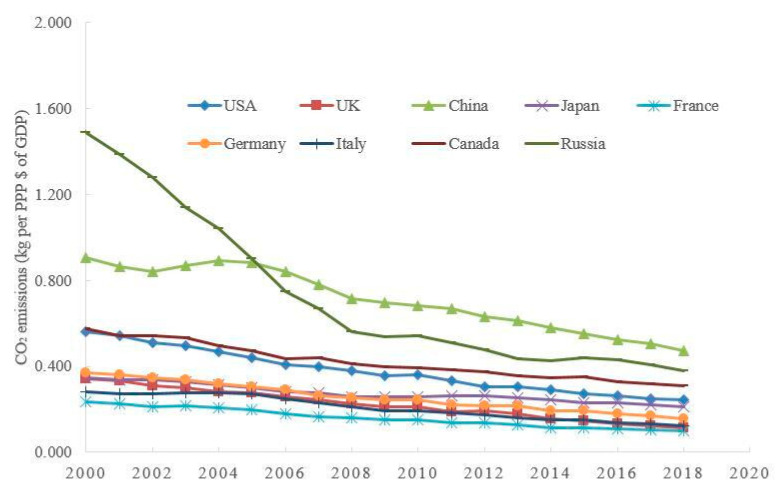
The trends of CO2 emissions per GDP of selected countries. Source: https://data.worldbank.org/indicator/EN.ATM.CO2E.PP.GD, accessed on 22 October 2022.

Many countries around the world have achieved outstanding progress in environmental governance. We can take the emission of greenhouse gases as an example. Figure 1 presents the trends of carbon dioxide (CO_2_) emissions per GDP of selected countries after the year 2000. These trends tell us that the carbon dioxide emissions per unit of GDP of these countries have been declining, not only because of improved traditional energy efficiency and the substitution of new energy sources, but also the efforts of various countries in environmental governance. Indeed, it is not just about carbon dioxide. With the increasing emphasis on environmental protection, the growth of emissions of different types of pollutants has slowed down [2]. Although global cooperation in environmental governance is affected by some political or economic factors, it has become a consensus that environmental governance can bring positive externalities to the economy and society.

**Figure 2:**
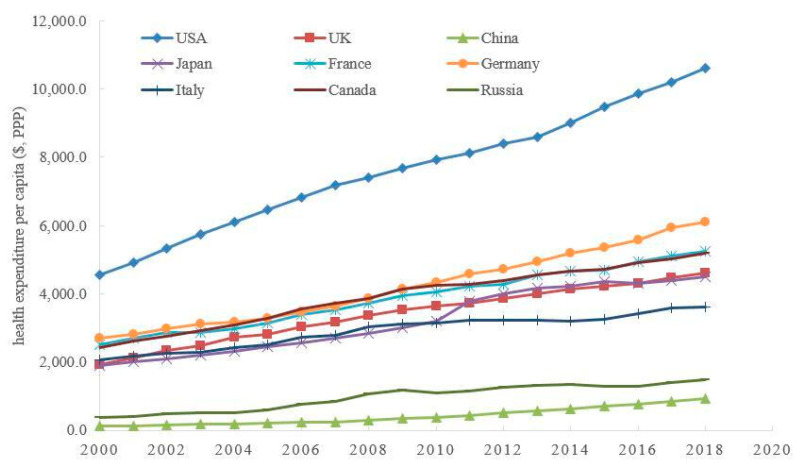
The trends of health expenditure per capita of selected countries. Source: https://data.worldbank.org/indicator/SH.XPD.CHEX.PP.CD, accessed on 22 October 2022.

Environmental governance will not only affect the emission of various pollutants, it may also have a positive influence on human health status. Consistent with the progress of environmental governance, the health status of human beings, on average, is becoming better than that of 20 years ago. Figure 2 shows the trends of health expenditure per capita of selected countries. The continual increase in health expenditure per capita reflects the growing importance these countries attach to the improvement of human health status, which provides the necessary support for meeting individuals’ health demands.

**Figure 3:**
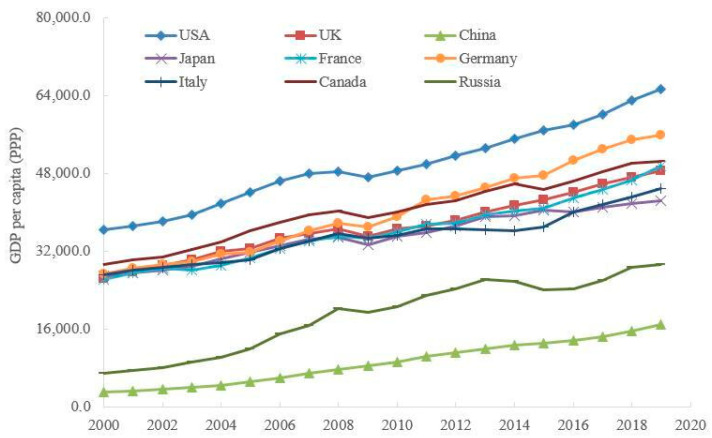
The trends of GDP per capita of selected countries. Source: https://data.worldbank.org/indicator/NY.GDP.PCAP.CD, accessed on 22 October 2022.

Meanwhile, the improvement of the health status can also create the foundation of human health capital for the long-term growth of the economy. In Figure 3, GDP per capita in the selected countries has been increasing over the past two decades, even though it experienced a decline temporally after the shock of the financial crisis in 2008.

From Figure 1, Figure 2 and Figure 3, it is not difficult to see that there is likely to be an endogenous relationship between the downward trend of greenhouse gas emissions, the upward trend of health expenditure, and economic growth. For example, China’s carbon dioxide emissions per unit of GDP are the highest in the selected countries, its health expenditure per capita is the lowest in the selected countries, and its GDP per capita is also the lowest in the selected countries. The above endogenous relationship seems to suggest that the reduction of pollutant emissions per capita (e.g., carbon dioxide emissions) may be a reason for the improvement of health, which may lay the foundation of healthy human capital for economic development. In addition, sustainable economic growth seemly also provides sufficient tax sources for environmental governance and public health improvement.

However, the endogenous relationship among environmental governance, public health, and economic growth may be more complex than that shown in Figure 1, Figure 2 and Figure 3. The reasons may be as follows. First, pollution emissions have a dual impact: on the one hand, pollution emissions reflect the short-term performance of the energy-dependent economy; on the other hand, the increasing pollution emissions aggravate the long-term policy costs of environmental regulation. Second, the health damage caused by pollution may also have dual effects: on the one hand, the health damage means that residents have to increase the cost of childcare for their children; on the other hand, government departments also need to increase the public health expenditure caused by pollution emissions. Although this endogenous relationship has attracted the attention of some scholars [3,4], there are still some outstanding issues worth pondering. First of all, under the irreversible trend of population aging, how will the above endogenous relationship change? Secondly, behind the balance between environmental pollution and economic growth, is there a balance between environmental governance and health improvement? Finally, when analyzing the effects of environmental policies, how do we evaluate the cost of these policies?

Therefore, this article attempted to incorporate environmental governance, health expenditures, and economic growth into an analytical framework to explore the laws underlying them, and propose corresponding policy enlightenments. We introduced the accumulation of health human capital and life expectancy into a general equilibrium analysis framework by constructing a three-period overlapping generation dynamic general equilibrium (OLG-DGE) model that included individual decision-making and corporate profits. This OLG-DGE model with optimized and balanced government budgets portrayed the internal connection among environmental governance, public health investment, and economic growth, which helped to lay the foundation for the later calibration analysis and policy simulation analysis. The research aimed to evaluate the adverse effects of environmental pollution on residents’ health status and economic growth, and then analyze the environmental effects, health effects, and economic growth effects of environmental governance and public health expenditure policies.

It is worth mentioning that the subsequent calibration analysis and policy simulation analysis in this paper was carried out in the context of China, and was mainly based on the following considerations. First, with the continuous development of China’s economy, the health damage caused by pollution makes people gradually face the trade-off between the quantity and quality of their children, which provided a realistic background for our analysis of environmental governance in the context of demographic transformation. Second, China implemented the most stringent environmental protection law in history in 2015 and further introduced a series of related environmental regulation laws in the following years (see Appendix A), which provided us with legal and institutional reference for analyzing the economic effects of environmental governance. Third, in addition to the introduction of relevant measures to control pollution emissions, these laws on environmental governance in China also emphasize the detection and improvement of public health caused by pollution, which provides valuable ideas for us to design environmental policy tools (e.g., environmental tax rate and public health expenditure proportion) in theoretical analysis.

Our findings in this paper can be generalized as follows. First, environmental governance policies may have an impact on economic output through the public health and life expectancy of residents despite their direct effect on the reduction of pollution emissions, which means that the rise of environmental tax does not always produce positive expected results. Second, we calibrated the OLG-DGE model through the China scenario, and the numerical analysis results well described the impact of environmental regulation policies on life expectancy, savings rates, and return on capital. Third, we conducted a policy simulation analysis based on the theoretical model, and the results confirmed the inverted U-shaped impact of the environmental tax rate on emission reduction and economic output. Fourth, the policy simulation also showed that the proportion of public expenditure devoted to improving the health damage caused by pollution emissions may have a trade-off between health improvement and economic output, which may depend on the level of environmental tax.

The rest of the paper is organized as follows. In Section 2, we present the related literature and point out the marginal contributions of this paper. In Section 3, we introduce the model, define an intertemporal equilibrium and make propositions about the equilibrium state. In Section 4, we calibrated the model through the China scenario and analyzed the socioeconomic effects of environmental pollution and control. In Section 5, we analyzed the effects of public policies on environmental governance and public health expenditure through policy simulation. Finally, in Section 6, we present the conclusions and policy implications.

## 2. Literature Review

Much literature has studied the impact of pollution on the health status of households. In general, the effects of pollution emissions on health can be divided into two categories. On the one hand, environmental pollution will directly affect the health of residents. Chen et al. studied the impact of air pollution on life expectancy based on the winter heating demarcation line of the Huai River in China; the results showed that 500 million residents in North China lost an average of five years of life expectancy compared to those in South China, due to severe air pollution [5]. Beatty and Shimshack studied the relationships between air pollution exposure and non-infant children’s respiratory health outcomes; their results indicated that increases in carbon monoxide and ground-level ozone are associated with statistically significant increases in children’s contemporaneous respiratory treatments [6]. It should be pointed out that not all environmental pollution is harmful to health, and mainly depends on the exposure level of the pollution emissions [7]. On the other hand, the adverse effects of pollution emissions on health may be transferred to the next generation through fertility. Currie et al. found consistently negative effects of exposure to carbon monoxide, both during and after birth; these results have important implications for the regulation of automobiles because they are the main source of carbon monoxide emissions [8]. Pons et al. estimated the effects of air pollution on birthweight; the results suggest a negative effect of PM2.5 on the lower tail of the weight distribution [9]. The intra- and inter-generational impacts of pollution emissions on health will change residents’ decisions on health investment and fertility. Therefore, the impact of environmental policies on intra- and inter-generational decision-making should not be overlooked when analyzing the health-improving effects of environmental governance [10,11,12].

The adverse effects of pollution emissions on health status also affect household decisions on health care, savings, education, pensions, and retirement at the micro level, which may influence economic growth and social welfare at the macro level. Gradus and Smulders regarded pollution emissions as a by-product of physical capital and introduced this factor into neoclassical growth theory in order to examine its impact on economic growth, which lays the foundation for later researchers to evaluate environmental taxation and environmental governance in the growth framework [13]. The environmental Kuznets curve (EKC) hypothesis posits an inverted U relationship between environmental pressure and per capita income, which is always used to reveal the impact of pollution emissions on economic growth [14,15,16,17]. However, the link between higher income and lower emissions implied by this inverted U relationship does not always exist [18]. Although fossil energy, as an important factor of production, is the driving force for economic growth, pollution emissions may hinder the sustainable growth of the economy: on the one hand, the increase in pollutants may be directly detrimental to economic growth because green environmental preferences are beneficial for growth and welfare improvements in the long run [19]; on the other hand, the adverse health effects of environmental pollution can affect labor supply and thus indirectly damage economic growth [20]. Therefore, environmental governance of pollution emissions may also affect economic growth through the above two paths. Aloi and Tournemaine found that a tighter environmental tax has positive effects on growth via two channels [21]: on the one hand, it will lead to improvement in workers’ health status and, thereby, productivity; on the other hand, it induces an optimization in resources reallocation towards R&D and, thereby, higher research intensity.

The adverse effects of pollution emissions on health and economic growth make environmental governance of more and more concern to theorists and policymakers. In recent years, many environmental policy tools have been proposed, such as environmental taxes, eco-taxes, tradable permits, voluntary agreements, and eco-labels [22,23,24,25]. Although the effects of these policy tools are confirmed in theoretical analysis, the influence of market-based policy instruments (such as emissions trading and taxes) in empirical studies may be weaker than assumed [26,27]. Environmental governance affects individuals’ intergenerational decisions on health risks and education, and promotes the optimal allocation of intergenerational resources; however, excessive policy costs in environmental governance may also adversely affect economic growth. Therefore, whether there is an optimal environmental tax rate in terms of environmental governance has attracted the attention of many scholars. Bovenberg and Mooij discussed the “double dividend” (not only a cleaner environment but also a less distortionary tax system) of environmental taxes in the general-equilibrium model, pointing out that the optimal environmental tax typically lies below the Pigovian tax in the presence of preexisting distortionary tax [28]. Compared with taking no measures, although the costs for environmental pollution control (i.e., investment in infrastructure and remedial measures) crowd out other public expenditures, this reduces the economic losses caused by environmental pollution and may lead to a higher GDP growth rate [29].

According to the existing literature, research on pollution control, public health, and economic growth has been quite rich. However, there is still a controversial issue worthy of studying further. First, due to the irreversibility of the aging trend, changes in population structure should not be ignored when analyzing the human health and economic growth effects of environmental governance. Gerlagh and van der Zwaan discussed the relationship among carbon emissions, population aging, and economic growth in an overlapping generational model (OLG) and provided a reference for analyzing demographic effects in environmental economics [30]. Wang et al. further pointed out that the inverted U-shape relationship reflected by the environmental Kuznets curve will be steeper and the peak will be higher when the population is growing positively [31]. Second, the optimal environmental tax rate should not only be limited to the trade-off between environmental governance costs and economic growth, but it should also fully consider the externality of environmental pollution on residents’ health. Since environmental pollution affects both health status at the micro level and economic output at the macro level, when we estimate the optimal environmental tax, we should take both health improvement and economic growth into account [4,32]. Third, although research based on empirical research can assess the effect of pollution control policies, the analysis of environmental policy costs and welfare improvement may depend on theoretical research. In particular, optimal environmental tax rates are often incorporated and calculated in the framework of general equilibrium analysis [33,34].

This article analyzed the relationship between environmental pollution (governance), public health, and economic growth in the OLG-DGE framework. In the model economy of this paper, we not only considered the optimal behavior of the household and production sectors, but also analyzed the micro- and macro-economic impacts of environmental governance policies (environmental taxes, abatement spending, and public health spending). Furthermore, in order to evaluate the health and economic effects of pollution emissions, the parameters in the model were calibrated by the actual economic conditions of China. We then assessed the impact of environmental governance policy tools on pollution emissions, health, life expectancy, and economic growth through policy simulations.

Compared with the existing literature, our work may be expansive in the following three ways. Firstly, considering that the health damage caused by environmental pollution may cause people to face a trade-off between the quantity and quality of their children, we introduced the family’s internal childcare cost into the OLG-DGE model when analyzing the economic impact of pollution regulation from the perspective of public health. Secondly, our model analysis of environmental pollution and environmental regulation was based on the Chinese scenario rather than being limited to simple numerical simulation, which was conducive to the policy reference for environmental regulation in those emerging economies with an aging trend. Thirdly, we also evaluated the pollution control effect of environmental regulation policy tools (environmental tax and public health expenditure ratio) and the policy cost in terms of health improvement and economic growth through policy simulation, which was helpful when providing policy implications for environmental regulation under multiple policy objectives.

## 3. Model Set Up

**Figure 4:**
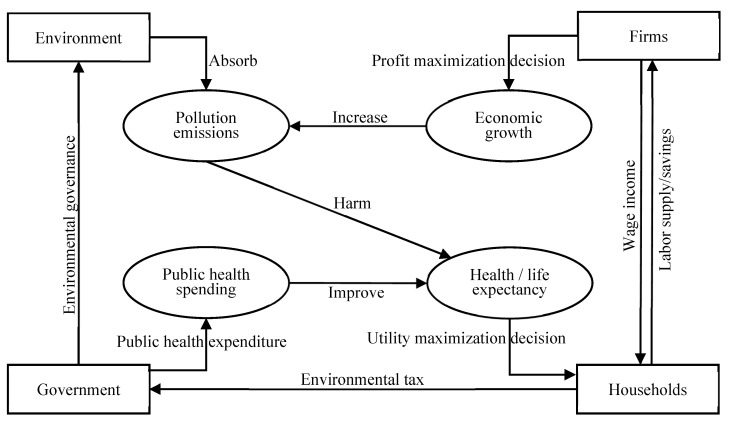
The logical framework of this article.

The logic framework of this article is represented in Figure 4. Specifically, we considered a three-period overlapping generation dynamic general equilibrium (OLG-DGE) model in this paper to depict the relationship between environmental governance, public health expenditure, and economic growth. Our framework included decisions of identical three-period lived households, profit maximization of representative companies, and a government that maintains a balanced budget. Specifically, the lifetime of an identical lived individual was divided into childhood, youth, and old age. We assumed that an individual is endowed with nothing and makes no economic decisions in childhood. The young people were endowed with one unit of labor time when they join the labor market. We also assumed that raising children is costly for the adult, and the number of children are exogenous (denoted as n). The cost of caring for children can then be assumed as $nqw_{t}$ (with nq∈(0,1)), where $w_{t}$ is the wage income per unit of labor [35,36,37]. When old, individuals retire and live with the amount of disposable income saved from t to *t* + 1.

### 3.1. Pollution Emissions and Health Status

Additionally, we assumed that individuals face a probability of surviving to old age. In particular, life expectancy in old age was assumed as endogenous, and crucially, even depends on health status denoted by $h_{t}$. In line with previous research [38,39,40,41,42,43,44,45], we assumed the following specific functional forms for survival probability in old age:

(1) $$\pi_{t+1}\left(h_{t}\right)=bh_{t}/\left(1+h_{t}\right)$$

where b∈(0,1) is an adjustment parameter to ensure that the expected life is within a reasonable range [43]. Therefore, $\pi_{t+1}\left(h_{t}\right)$ is increasing from 0 to b and is strictly concave with $h_{t}$. As $\pi \left(h_{t}\right)$ reflects the probability of individuals living through the entire old-age period, then we call this life expectancy, longevity, or survival probability interchangeably.

Pollution emissions and public health expenditure are two important factors for the health status of the household [30,43,46]. In addition, individuals’ health status depends positively on the parent’s health status to some extent [47,48,49]. Therefore, the health human capital of an adult individual in period *t*+1 was assumed as follows:

(2) $$h_{t}=\left(1-\mu \right)h_{t-1}+H{\left(g_{t}/P_{t}\right)}^{\sigma }$$

where $h_{t-1}$ denotes the parent’s health status,$\;g_{t}$ denotes public health expenditure, and $P_{t}$ denotes pollution emissions. The parameter μ∈(0,1) reflects the intensity of the impact from the parents’ health status on the next generation’s health status (which can also be regarded as the depreciation rate for health status), σ denotes the output elasticity of $g_{t}/P_{t}$ with respect to health status, and H  > 0 denotes technology for the accumulation of health status.

### 3.2. Optimization of Utility for Individuals

According to the assumptions above, we can obtain the relationship between the young and the old in the same period. Specifically, assuming there are $N_{t}^{y}$ and $N_{t+1}^{y}$ young individuals in period *t* and period *t* + 1, respectively. As we assume that each young person gives birth to $n_{t}$ children, then we have $N_{t+1}^{y}=nN_{t}^{y}$. The number of old individuals in period *t* + 1 is assumed as $N_{t+1}^{o}$; hence, $N_{t+1}^{o}=\pi \left(h_{t}\right)N_{t}^{y}$. Therefore, the old-age dependency ratio, regarded as the fraction of the old to the young, in this model economy is given by [50]:

(3) $$\frac{N_{t+1}^{o}}{N_{t+1}^{h}}=\pi_{t+1}\left(h_{t}\right)/n$$

In line with the assumption of intergenerational altruism [51,52], the representative individual derives utility (U) from his or her adulthood consumption $c_{t}^{y}$, elderhood consumption $c_{t+1}^{o}$, and the number of his or her children n. Therefore, the representative individual at period *t* makes decisions on consumptions and fertility to maximize the lifetime utility function:

(4) $$U=lnc_{t}^{y}+\beta \pi_{t+1}\left(h_{t}\right)lnc_{t+1}^{o}$$

where $c_{t}^{y}$ and $c_{t+1}^{o}$ represent the consumption in the young period and the old period, respectively. The parameter β∈(0,1) is the degree of individuals’ patience to consume over the life cycle. The budget constraints for period *t* and *t* + 1 are the following:

(5) $$c_{t}^{y}+s_{t}=w_{t}\left(1-nq\right)\left(1-\tau_{p}\right)$$

(6) $$\pi_{t+1}\left(h_{t}\right)c_{t+1}^{o}=s_{t}R_{t+1}$$

where $s_{t}$ is the savings of the young in the period *t*, $w_{t}$ is the wage of the adult, and q represents the proportion of the monetary cost of rearing a child within taxed income. In addition,$\;\tau_{p}$ ∈(0,1) is an environment tax rate that finances any kind of public expenditure related to environmental governance. Moreover, we assumed that intermediaries in financial markets operate in a perfectly competitive market and the corresponding rate must incorporate the risk caused by the uncertainties of agents’ lifetimes. Hence, the rate of return on savings is $R_{t+1}/\pi \left(h_{t}\right)$, where $R_{t+1}$ is the risk-free interest rate [39,53].

According to the above settings, maximization of utility subject to the budget constraints gives the first-order conditions (F.O.C.) of $c_{t}^{y}$ and $c_{t+1}^{o}$ as follows:

(7) $$c_{t+1}^{o}=\beta R_{t+1}c_{t}^{y}$$

Combining Equations (5)–(7), we can obtain the solutions of $c_{t}^{y}$, $s_{t}$ and $n_{t}$:

(8) $$c_{t}^{y}=\frac{1}{1+\beta \pi_{t+1}\left(h_{t}\right)}w_{t}\left(1-nq\right)\left(1-\tau_{p}\right)$$

(9) $$s_{t}=\frac{\beta \pi_{t+1}\left(h_{t}\right)}{1+\beta \pi_{t+1}\left(h_{t}\right)}w_{t}\left(1-nq\right)\left(1-\tau_{p}\right)$$

### 3.3. Production

The output market was assumed to be perfectly competitive and the production function was defined as Cobb–Douglas forms: $Y_{t}=AK_{t}^{\alpha }L_{t}^{1-\alpha }$, where A>0, and α∈(0,1) reflects the elasticity. By assuming perfect competition, the profit maximization problem remained the same in each period:

(10) $$Max\left\{AK_{t}^{\alpha }L_{t}^{1-\alpha }-R_{t}K_{t}-w_{t}L_{t}\right\}$$

Defining $y_{t}=Y_{t}/L_{t}$ denotes the output per labor, and $k_{t}=K_{t}/L_{t}$ denotes the capital per labor. The intensive production function can then be written as:

(11) $$\;y_{t}=Ak_{t}^{\alpha }$$

Therefore, profit maximization yields:

(12) $$R_{t}=\alpha Ak_{t}^{\alpha -1}$$

(13) $$w_{t}=\left(1-\alpha \right)Ak_{t}^{\alpha }$$

### 3.4. Public Sector

The government finances health investments and environmental protection at a balanced budget by levying on output. In particular, the government collects revenues through a tax rate ($\;\tau_{p}$) on wage income, which is the source of health expenditure and environmental protection. Then, the environmental tax per labor unit is $\tau_{p}w_{t}\left(1-nq\right)$, which is divided into two parts to provide public services. One is used for public health expenditure (noted as $g_{t}$) for preventing disease caused by pollution, including investment in health infrastructure and public health services. The other is used for pollution emissions elimination (noted as $m_{t}$) in order to protect the environment from damaging effects induced by pollution. Therefore, the condition of the government budget balance can be written as:

(14) $$\tau_{p}w_{t}\left(1-nq\right)=g_{t}+m_{t}$$

Assuming that ϕ is the fraction of public health expenditure in the total public expenditure, then:

(15) $$g_{t}=\phi \tau_{p}w_{t}\left(1-nq\right)$$

(16) $$m_{t}=\left(1-\phi \right)\tau_{p}w_{t}\left(1-nq\right)$$

As is standard in the literature [54,55,56,57,58,59], the pollution emissions per labor unit in period *t* + 1 (noted as $P_{t}$) are determined by the function below:

(17) $$P_{t+1}=\left(1-\delta \right)P_{t}+\rho y_{t}-\chi m_{t}$$

The pollution emissions per labor unit in period t (namely $P_{t}$ in Equation (17)) can be understood as the stock of pollution inherited from the previous period. The parameter δ∈(0,1) denotes the natural rate of pollution absorption, ρ≥ 0 reflects the degree of pollution induced by production, and χ≥ 0 captures the efficiency of pollution emissions elimination caused by environmental governance. It should be further explained that the two parameters ρ and χ have rich economic implications: the former reflects the degree of economic dependence on energy consumption, and the latter reflects the degree of technological innovation in the field of energy utilization. In order to ensure the effectiveness of pollution control [60,61], our further analysis was based on the following assumption.

**Assumption** **1:**
ρ *<* χ *. This assumption means every* ρ *unit pollution caused by per unit output at least can be eliminated by one unit expenditure of environmental protection.*

### 3.5. General Equilibrium

The intertemporal equilibrium for this economy was defined by a set of sequences satisfying all equilibrium conditions. Specifically, if the initial physical capital $K_{0}$, pollution emissions $P_{0},$ and health status $h_{0}$ are given, the competitive equilibrium for this economy can be defined as follows:

(i) Individuals maximize utility.
(ii) Firms maximize profits.
(iii) Labor market clear.
  (18) $$L_{t}^{D}=N_{t}^{y}$$
(iv) Capital market clear. $K_{t+1}=N_{t}^{y}s_{t}$
  (19) $$nk_{t+1}=s_{t}$$
(v) Government satisfies budget balance.
(vi) Environment quality satisfies Equation (17).

### 3.6. Policy Implications

To identify the solutions for this model economy, we substituted Equations (13) and (16) into Equation (17) and obtained:

(20) $$P_{t+1}=\left(1-\delta \right)P_{t}+\rho Ak_{t}^{\alpha }-\chi \left(1-\phi \right)\tau_{p}\left(1-nq\right)\left(1-\alpha \right)Ak_{t}^{\alpha }$$

Equation (19) reflects the dynamics of pollution emissions from period *t* to period *t* + 1. Similarly, substituting Equations (10), (11), and (13)–(16) into (19), we obtained the dynamics of capital per labor unit from period t to period *t* + 1:

(21) $$k_{t+1}=\frac{\beta \pi_{t+1}\left(h_{t}\right)}{1+\beta \pi_{t+1}\left(h_{t}\right)}\left(1-nq\right)\left(1-\tau_{p}\right)\left(1-\alpha \right)Ak_{t}^{\alpha }$$

A steady state was defined by $h_{t}=h_{t-1}=h^{*}$, $P_{t+1}=P_{t}=P^{*},$ and $k_{t+1}=k_{t}=k^{*}$ for all *t*. Substituting these three equations into Equations (2), (20), and (21), we demonstrated the existence and uniqueness of a stationary solution ($k^{*}$,$\;P^{*}$, $h^{*}$). Then:

(22) $$k^{*}={\left\{\frac{A\beta bH\delta^{\sigma }\phi^{\sigma }{\left(\tau_{p}\right)}^{\sigma }\left(1-\tau_{p}\right){\left(1-nq\right)}^{1+\sigma }{\left(1-\alpha \right)}^{1+\sigma }}{n\mu {\left[\rho -\chi \left(1-\phi \right)\tau_{p}\left(1-nq\right)\left(1-\alpha \right)\right]}^{\sigma }+n\left(1+\beta \right)H\delta^{\sigma }{\left[\phi \tau_{p}\left(1-nq\right)\left(1-\alpha \right)\right]}^{\sigma }}\right\}}^{1/\left(1-\alpha \right)}$$

(23) $$P^{*}=\frac{1}{\delta }\left[\rho -\chi \left(1-\phi \right)\tau_{p}\left(1-nq\right)\left(1-\alpha \right)\right]A{\left(k^{*}\right)}^{\alpha }$$

(24) $$h^{*}=\frac{H}{\mu }{\left[\phi \tau_{p}\left(1-nq\right)\left(1-\alpha \right)A{\left(k^{*}\right)}^{\alpha }\right]}^{\sigma }$$

According to Equation (23), in order to ensure $P^{*}>0$, our analysis should follow Assumption 2.

**Assumption** **2:**
$\rho -\chi \left(1-\phi \right)\tau_{p}\left(1-nq\right)\left(1-\alpha \right)$ *> 0. This assumption suggests that* $\tau_{p}<\rho /\left[\chi \left(1-\phi \right)\left(1-nq\right)\left(1-\alpha \right)\right]$ *.*

From Equation (22) we can see that $k^{*}\to 0$ if $\tau_{p}\to 0$ or $\tau_{p}\to 1$, which implies the existence of an optimal tax rate (named as $\tau_{p}^{m}$) for capital per labor unit. In addition, according to Equations (12), (23), and (24), $y^{*}$, $P^{*}$ and $h^{*}$ are monotonically increasing functions of $k^{*}$, which suggests that $y^{*}\to 0$, $P^{*}\to 0,$ and $h^{*}\to 0$ if $\tau_{p}\to 0$ or $\tau_{p}\to 1$. Therefore, we can also find the optimal environmental tax rate between 0 and 1 that maximizes output per labor unit, pollution emissions per labor unit, and health status per labor unit. As capital per labor unit in the conditions of equilibrium is the basis for calculating other endogenous variables (e.g., $y^{*}$, $P^{*}$ and $h^{*}$), we next focused on the analysis of optimal policy tools (e.g., $\tau_{p}$ and ϕ) for $k^{*}$. By applying Assumption 2 to Equation (22), we created the following proposition.

**Proposition** **1:** *If* ρ/[χ(1−ϕ)(1−nq)(1−α)]≥1 *and* $\tau_{p}^{m}\in \left(0,1\right)$ *, then there must be an optimal environmental tax rate that maximizes capital per labor unit; however, in the condition of* [χ(1−ϕ)(1−nq)(1−α)]<1 *, only if* $0<\tau_{p}^{m}<\left[\chi \left(1-\phi \right)\left(1-nq\right)\left(1-\alpha \right)\right]$ *there exists an optimal environmental tax rate that maximizes output per labor unit.*

**Proof:**  Let $\frac{\partial k^{*}}{\partial \tau_{p}}=0$, then we have:$\;F\left(\tau_{p}\right)=0$. By solving this equation we can obtain the optimal environmental tax rate $\tau_{p}^{m}$. We can see that the relationship between $\tau_{p}^{m}$ and ρ/[χ(1−ϕ)(1−nq)(1−α)] is determined by the exogenous parameters. Therefore, the optimal environmental tax rate does not always exist. □

From Proposition 1, we highlight that the existence of an optimal environmental tax rate is based on some conditions. Proposition 1 also suggests that we can obtain the optimal environmental tax rate with respect to $y^{*}$, $P^{*},$ and $h^{*}$ through a similar method. It should be noted that the optimal environmental tax rate for $k^{*}$, $y^{*}$, $P^{*},$ and $h^{*}$ may not be the same. Hence, the government may be faced with trade-offs when changing the environmental tax rate.

The fraction of public health expenditure ϕ is another policy tool in this model economy. By applying Assumption 2 to Equation (22), we have the following proposition:

**Proposition** **2:** *If* $0<\tau_{p}<\rho /\left[\chi \left(1-nq\right)\left(1-\alpha \right)\right]$ *,* $\frac{\partial k^{*}}{\partial \phi }<0$ *, which means* $k^{*}$ *reaches its maximization in the condition of* ϕ→1 *; if* $\rho /\left[\chi \left(1-nq\right)\left(1-\alpha \right)\right]<\tau_{p}<1$ *,* $\frac{\partial k^{*}}{\partial \phi }<0$ *, which means* $k^{*}$ *reaches its maximization in the condition of* ϕ→0 *.*

**Proof:**  Defining $\frac{\partial k^{*}}{\partial \phi }=F\left(\tau_{p}\right)$, then sign of $F\left(\tau_{p}\right)$ depends on $\rho -\chi \tau_{p}\left(1-nq\right)\left(1-\alpha \right)$. □

Proposition 2 states that the increase in the proportion of public health expenditure will not always improve the capital per labor unit. Similarly, we can generalize Proposition 2 from $k^{*}$ to $y^{*}$, $P^{*},$ and $h^{*}$, as these endogenous variables are monotonically increasing functions of $k^{*}$.

Moreover, as the survival probability for the old is a monotonically increasing functions of $h^{*}$, we substituted Equations (12), (15), (22), and (23) into Equation (1) and obtained the endogenous survival probability $\pi \left(h^{*}\right)$:

(25) $$\pi \left(h^{*}\right)=\frac{b\delta^{\sigma }H{\left[\phi \tau_{p}\left(1-nq\right)\left(1-\alpha \right)\right]}^{\sigma }}{\mu {\left[\rho -\chi \left(1-\phi \right)\tau_{p}\left(1-nq\right)\left(1-\alpha \right)\right]}^{\sigma }+H{\left[\phi \tau_{p}\left(1-nq\right)\left(1-\alpha \right)\right]}^{\sigma }}$$

Policy parameters $\tau_{p}$ and θ in Equation (26) reflect the role that government plays in life expectancy. By applying Assumption 2 to Equation (25), we have the following proposition:

**Proposition** **3:** *As* $\frac{\partial \pi^{*}}{\partial \tau_{p}}>0$ *, the life expectancy will be increased by rising the environmental tax rate. However, if* $0<\tau_{p}<\rho /\left[\chi \left(1-nq\right)\left(1-\alpha \right)\right]$ *, then* $\frac{\partial \pi^{*}}{\partial \phi }>0$ *, which means the life expectancy will be increased by raising the fraction of public health expenditure; if* $\rho /\left[\chi \left(1-nq\right)\left(1-\alpha \right)\right]<\tau_{p}<1$ *, then* $\frac{\partial \pi^{*}}{\partial \phi }\leq 0$ *, which means the life expectancy will be decreased by raising the fraction of public health expenditure.*

**Proof:**  By applying Assumption 2 to Equation (25), we can easily prove that $\frac{\partial \pi^{*}}{\partial \tau_{p}}>0$. However, the sign of $\frac{\partial \pi^{*}}{\partial \phi }$ then depends on $\rho -\chi \tau_{p}\left(1-nq\right)\left(1-\alpha \right),$ according to Equation (25). □

Proposition 3 suggests that the government can affect life expectancy by changing the environmental tax rate. Therefore, environmental governance is conducive to controlling the adverse effect of pollution emissions on life expectancy. However, public health expenditure will not always improve the survival probability of old individuals. From Proposition 3 we highlight that the increase in the fraction of public health expenditure will improve life expectancy only under the condition of a lower environmental tax rate ($0<\tau_{p}<\rho /\left[\chi \left(1-nq\right)\left(1-\alpha \right)\right]$), and that the increase in the fraction of environmental protection expenditure will improve life expectancy under the condition of a higher environmental tax rate ($\rho /\left[\chi \left(1-nq\right)\left(1-\alpha \right)\right]<\tau_{p}<1$).

We also should note that the saving rate is endogenously determined in this economy because of the endogenous survival probability. According to Equation (10), the saving rate is defined as ${\hat{s}}_{t}=s_{t}/\left[w_{t}\left(1-\tau_{p}\right)\right]=\frac{\beta \pi_{t+1}\left(h_{t},e_{t}\right)\left(1-nq\right)}{1+\beta \pi_{t+1}\left(h_{t},e_{t}\right)}$. Therefore, we can obtain the saving rate by substituting Equation (25) into this equation:

(26) $${\hat{s}}^{*}=\frac{\beta b\delta^{\sigma }H\left(1-nq\right){\left[\phi \tau_{p}\left(1-nq\right)\left(1-\alpha \right)\right]}^{\sigma }}{\mu {\left[\rho -\chi \left(1-\phi \right)\tau_{p}\left(1-nq\right)\left(1-\alpha \right)\right]}^{\sigma }+\left(1+\beta b\delta^{\sigma }\right)H{\left[\phi \tau_{p}\left(1-nq\right)\left(1-\alpha \right)\right]}^{\sigma }}$$

From Equation (26) we can see that the saving rate is also determined by government intervention in environmental protection and public health (policy parameters $\tau_{p}$ and ϕ). Then, we have the following proposition:

**Proposition** **4:** *As* $\frac{\partial {\hat{s}}^{*}}{\partial \tau_{p}}>0$ *, a rise in the environmental tax rate will increase the saving rate. However, if* $0<\tau_{p}<\rho /\left[\chi \left(1-nq\right)\left(1-\alpha \right)\right]$ *, then* $\frac{\partial \pi^{*}}{\partial \phi }>0$ *, which means a rise in the fraction of public health expenditure will increase the saving rate; if* $\rho /\left[\chi \left(1-nq\right)\left(1-\alpha \right)\right]<\tau_{p}<1$ *, then* $\frac{\partial \pi^{*}}{\partial \phi }\leq 0$ *, which means a rise in the fraction of public health expenditure will decrease the saving rate.*

**Proof:**  By applying Assumption 2 to Equation (26), we can easily prove that $\frac{\partial {\hat{s}}^{*}}{\partial \tau_{p}}>0$. However, the sign of $\frac{\partial {\hat{s}}^{*}}{\partial \phi }$ then depends on $\rho -\chi \tau_{p}\left(1-nq\right)\left(1-\alpha \right),$ according to Equation (26). □

Proposition 4 states that the government can also affect private saving rates by changing the environmental tax rate. As private savings are an important source of physical capital, Proposition 4 can be regarded as a micro-explanation for the impact of environmental tax rates on physical capital per labor unit (proposed in Proposition 1). However, public health expenditure will not always increase the private saving rate. From Proposition 3, we highlight that the increase in the fraction of public health expenditure will increase the private saving rate only under the condition of a lower environmental tax rate ($0<\tau_{p}<\rho /\left[\chi \left(1-nq\right)\left(1-\alpha \right)\right]$), and that the increase in the fraction of environmental protection expenditure will increase the private saving rate only in the condition of a higher environmental tax rate ($\rho /\left[\chi \left(1-nq\right)\left(1-\alpha \right)\right]<\tau_{p}<1$). Therefore, Proposition 4 can be regarded as a micro-explanation for the impact of the fraction of public health expenditure on physical capital per labor unit proposed in Proposition 2.

## 4. Numerical Simulation

### 4.1. Parameter Calibration

We derived a full numerical solution for the model. The objective was to illustrate the dynamics of the endogenous variables (e.g., growth, life expectance, and fertility) when considering pollution emissions and environmental governance. Before proceeding with the simulated analysis of the model, we parameterized the model in order to reflect the real situations in China. The calibrated parameters in the base case are summarized in Table 1.

1. Calibrations for economic growth. According to Qiao and Wang [62], the annual total factor productivity of China is assumed as 4.5%, then A = (1 + 4.5%)^30^ = 3.7453. As the share of capital in GDP is about 0.4–0.6 in China [63,64], we set α = 0.60 in the base case.
2. Calibrations for household decisions. Each period in this model was assumed to be 30 years; then the time-discount factor *β* was calibrated to match the empirically observed saving rate in 2020, which required β = 0.9930. Considering that the total child-rearing cost for a couple consists of childbirth costs, childcare costs, and education costs, then the percentage of total child-rearing costs on working income is higher than the proportion of education costs in Chinese households. According to the China Statistical Yearbook 2021, residents’ private education expenditure in 2020 was about 6.3% of total consumption expenditure [65]. The parameter *q* was then assumed as *q* = 0.12.
3. Calibrations for health and population structure. Considering the limited impact on health from the genetic factors of parents, we assumed that the depreciation rate for health status accumulation was μ = 85%, and the output elasticity for health status accumulation was σ = 0.5. The technology parameter for health status accumulation was assumed to be the same as total factor productivity, namely *H* = *A*. The total fertility rate in China is about 1.3 according to the 7th National Census of China in 2020 [66]; hence, we assumed that n = 0.65 to reflect the number of children per adult. The adjustment parameter for life expectance was set as b = 0.88 in order to match the real-life expectancy in 2020 of China.
4. Calibrations for pollution emissions. In this model economy, pollution emissions were assumed as the key point for public health and life expectance. Therefore, the parameters for pollution emissions were calibrated in order to meet the real-life expectancy in China. Considering the diversity of the natural rate of absorption on different pollutants, we assumed that the average annual natural rate of pollution absorption was 10% each year. Thus, δ = 1 − (1 − 10%)^30^ ≈ 0.96 in the base case. In a resource-driven economy, pollution emissions tend to be positively correlated with economic output. Therefore, we assumed the degree of pollution induced by production ρ = 0.06 in the base case. In addition, as public spending on pollution abatement reduces pollution emissions, we assumed the efficiency of pollution elimination χ  = 0.85 in the base case.
5. Calibrations for policy parameters. Although the Environmental Protection Tax Law of the People’s Republic of China was not implemented until 2018 (see Appendix A), public expenditure on environmental governance can be traced back to the end of the last century. For example, the 1999 China Statistical Yearbook reported on the punishment and compensation for environmental pollution, which was the composition of public revenue and expenditure. Therefore, the introduction of an environmental tax rate and the fraction of public health expenditure into this OLG-DGE model economy and the calibration under the Chinese scenario could not only explain the direct effect and economic impact of China’s environmental regulations, it could also help to further predict the long-term costs of environmental regulation policies in public health and economic growth. The above two policy parameters can be calibrated through real expenditure in environmental governance and public health in China. According to a statistical report released by the National Bureau of Statistics of China [67], the Gross Domestic Product (GDP) of China in 2020 was RMB 101,598.6 billion. According to data released by the Ministry of Finance of China [68], China’s environmental protection spending in 2020 was RMB 631.7 billion, and public health spending was RMB 1920.1 billion. According to data released by the National Healthcare Security Administration of China [69], the medical insurance spending of China in 2020 was RMB 2103.2 billion. Therefore, the environmental tax rate $\tau_{p}$ = (6317 + 1920.1 + 2103.2)/101,598.6 ≈ 0.046, and the fraction of public health expenditure ϕ = 1 − 6317/(6317 + 19,201 + 21,032) ≈ 0.86.

### 4.2. Baseline Analysis

According to the parameter values in the benchmark case reported in Table 1, the endogenous variables in the model economy can be numerically analyzed. The endogenous life expectancy (probability of survival in the elderly) was calculated according to Equation (25). The endogenous savings rate was calculated according to Equation (26). We calculated the endogenous capital rate by substituting Equation (22) into Equation (12), which gave the annualized interest rate. The numerical analysis results of the above three endogenous variables are reported in Table 2. To test the accuracy of the numerical results, the actual values of the endogenous variables in China are also shown in Table 2.

1. Life expectancy. According to data released by the State Council of China, life expectancy in China in 2020 was 77.93 years [70]. The real survival probability for old age was (77.93 − 60)/30 ≈ 0.5976. The simulated survival probability value for old age was 0.5950 in this model economy. By comparing these two values, the absolute error of life expectancy was only −0.0026, and the relative error was within −1%.
2. Savings rate. As the household consumption expenditure was shocked by the COVID-19 epidemic, 2019 data was used to calculate the real household saving rate. According to the China Statistical Yearbook 2020, residents’ disposable income and private total consumption expenditure in 2020 were RMB 30732.8 and RMB 21558.9, respectively. Therefore, the savings rate in 2020 was calculated as 1 − 21558.9/30732.8 ≈ 0.2985. The simulated value for the savings rate was 0.2857 in this model economy. By comparing these two values, the absolute error of life expectancy was only −0.0128, and the relative error was −4.29%.
3. Annual capital rate. The 5-year loan prime rate (LPR), a reference for the long-term benchmark interest rate, was 4.65% in 2020, according to data released by the People’s Bank of China. The simulated value for the annual capital rate was 4.45% in this model economy. By comparing these two values, the absolute error of life expectancy was only −0.20%, and the relative error was −4.30%. Indeed, the 5-year LPR after May 2022 has dropped to 4.45%, which is very close to the numerical analysis result of the model.

### 4.3. Socio-Economic Effects of Pollution Emissions and Control

On the basis of the above-mentioned benchmark analysis, the following is an in-depth analysis of the socio-economic effects of pollution emissions and environmental governance. These socioeconomic effects can be divided into two aspects: one is health effects, including changes in health status and life expectancy; the other is economic effects, including changes in savings rates and outputs. As health status, life expectancy, savings rates, and outputs per labor unit were endogenously determined by the model, changes in the degree of pollution emissions and environmental governance efficiency will affect these variables.

**Figure 5:**
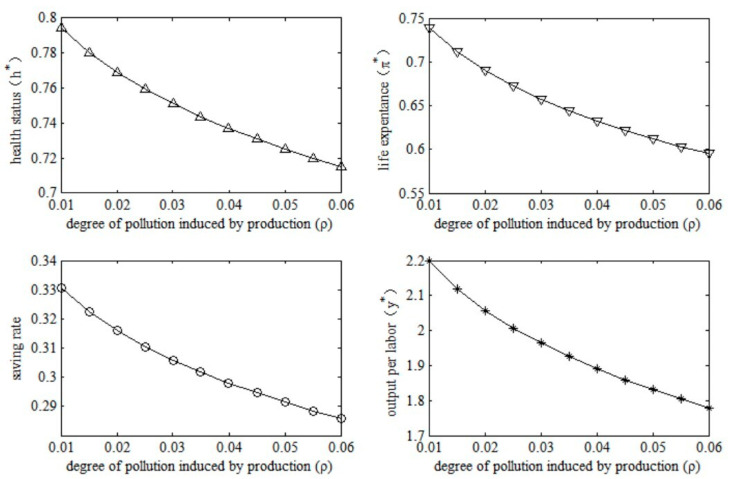
Socioeconomic effects of degree of pollution induced by production. Note: * of this figure represents the numerical solutions of these variables in equilibrium. The same is as below.

1. Socio-economic effects of pollution emissions. In a resource-driven economy, the achievement of economic growth goals will inevitably result in an increase in pollution emissions. With economic transformation and technological progress, energy efficiency will increase, which will lead to a decrease in pollution emissions per unit of output. Therefore, we examined the socioeconomic effects of changes in the degree of pollution induced by production by changing the parameter *ρ* in this model economy. The results are shown in Figure 5.

The results in Figure 5 indicate that the increase in the degree of pollution induced by production will have negative socioeconomic effects. Specifically, health conditions will worsen after the increase in the degree of pollution induced by production. The deterioration in health will then reduce the probability of survival in old age, which means a lower life expectancy. In terms of economic effects, the increase in health risks in old age will lead to a decline in the saving rate, which implies that people are reluctant to save enough for the old-age period. A fall in saving means that the accumulation of physical capital would be impeded, and therefore output per unit of labor would fall. It can be seen that the increase in pollution emissions not only leads to increased health risks at the micro level, but also hinders macroeconomic growth.

**Figure 6:**
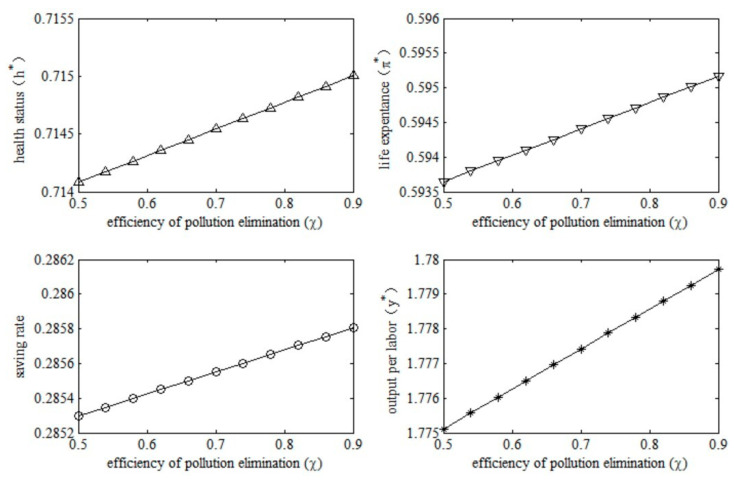
Socioeconomic effects of pollution elimination efficiency.

2. Economic effects of pollution elimination efficiency. In an environment-friendly economy, the efficiency of environmental governance has attracted much attention. With the improvement of pollution elimination efficiency, pollution emissions will change. Therefore, we examined the socioeconomic effects of changes in pollution elimination efficiency by changing the parameter χ in this model economy. The results are shown in Figure 6.

The results in Figure 6 indicate that the increase in pollution elimination efficiency will have positive socioeconomic effects. Specifically, health conditions will improve after the increase in the efficiency of pollution elimination. As a result, the improvement in health status will increase the probability of survival in old age, which means a longer life expectancy. In terms of economic effects, the longer life expectancy in old age will lead to an increase in the saving rate, which implies that people tend to save enough for old age. An increase in saving means a rapid accumulation of physical capital, and therefore output per unit of labor will also increase. It can be seen that the increase in pollution elimination efficiency will not only lead to lower health risks at the micro level, it will also lead to higher levels of macroeconomic output.

Moreover, by comparing Figure 5 and Figure 6, we can see that the socioeconomic effects of the degree of pollution induced by production are greater than that of pollution elimination efficiency when the marginal changes of the two are equal. This result shows that it is better to reduce the pollution emissions per unit of output or improve the utilization efficiency of resources (energy) than to dedicate to environmental governance after the environment is polluted and deteriorated.

## 5. Further Discussion: Policy Simulation

In the model economy of this paper, there are two policy tools of environmental governance, namely the environmental tax rate and the fraction of public health expenditure. These two policy tools reflect the financing sources of environmental governance and the preference for environmental governance expenditures, respectively. When analyzing the policy effects of environmental governance, we focus on the impacts on pollution emissions, health status, life expectancy, and economic output.

### 5.1. Changes in Environmental Tax Rate

**Figure 7:**
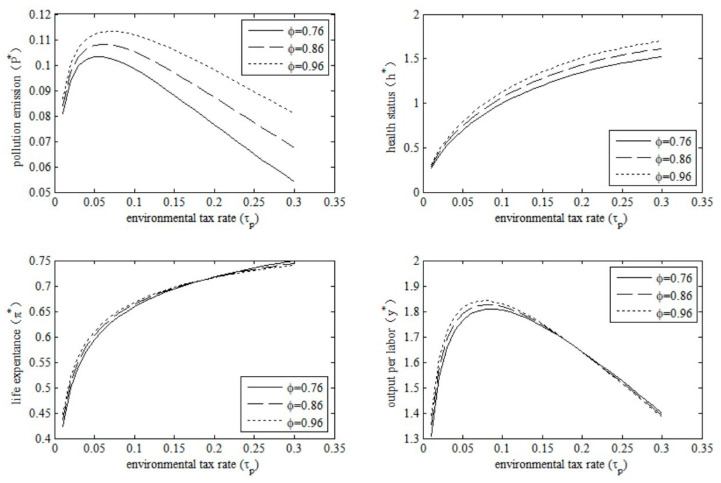
Policy effects of the environmental tax rate.

The collection of environmental taxes is a commonly used environmental governance measure by the government. However, government departments have to take into account the multiple goals of environmental governance, health improvement, and economic growth, which may lead to differences in the impact of environmental taxes from the micro and macro perspectives. Therefore, we examined the policy effects of changes in the environmental tax rate by changing the policy parameter $\tau_{p}$ in this model economy. The results are shown in Figure 7.

The results in Figure 7 show that the policy effects of the increase in the environmental tax rate on different endogenous variables differ. Specifically, the impact of environmental taxes on pollution emissions is inverted U-shaped. Thus, simply increasing environmental taxes will not necessarily reduce pollution emissions. The effects of environmental taxes on health and life expectancy are monotonic. An increase in environmental taxes would improve health, and health improvements would increase life expectancy in old age. Additionally, when the environmental tax rate is low (e.g., $\tau_{p}\;$ < 0.05), an environmental tax rise may improve the health situation and stimulate economic growth; however, it cannot effectively curb the increase in pollution emissions. Moreover, combined with the four graphs in Figure 7, we can hold that an excessive environmental tax rate (e.g., $\tau_{p}\;$ > 0.1) can reduce pollution emissions, and improve health risks and life expectancy, but at the expense of economic growth. Therefore, in order to reduce pollution emissions, environmental taxes should be kept within an appropriate range.

### 5.2. Changes in Fraction of Public Health Expenditure

**Figure 8:**
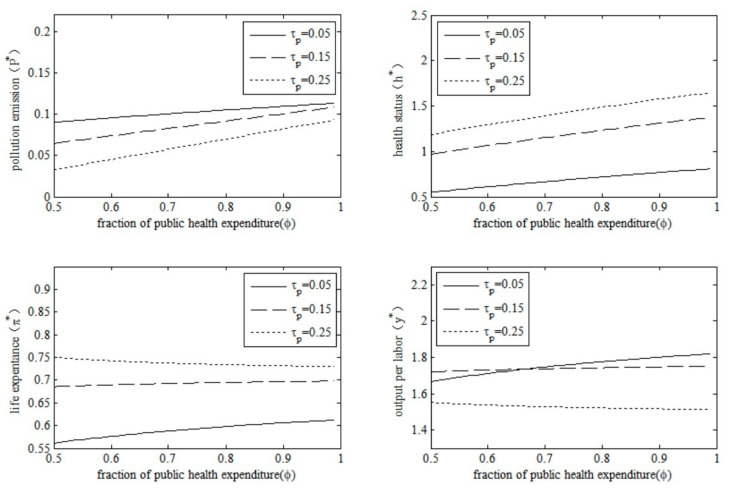
Policy effects of the fraction of public health expenditure.

Expenditure preferences are another policy tool dedicated to environmental governance. Environmental governance decisions are likely to be contested between eliminating pollution and improving health risks, which means that emphasizing one will ignore the other. Therefore, we examined the policy effects of changes in the fraction of public health expenditure by changing the policy parameter ϕ in this model economy. The results are shown in Figure 8.

The results in Figure 8 show that the policy effects of the increase in the fraction of public health expenditure on different endogenous variables also differ. Specifically, the pollution emissions will increase as the fraction of public health expenditure increases, due to less share of spending on pollution abatement. Additionally, although an increased share of health spending improves health, it does not necessarily lead to an increase in life expectancy in old age. In particular, when the environmental tax rate is low (e.g., $\tau_{p}\;$ = 0.05), an increase in the fraction of public health expenditure will increase health status as well as life expectancy; however, when the environmental tax rate is high enough (e.g., $\tau_{p}\;$ = 0.25), the increase in the fraction of public health expenditure will decrease life expectancy, which may be due to the increase in pollution emissions, which will be faster than the increase in health improvement. Furthermore, an increase in the fraction of public health spending will not necessarily lead to an increase in the level of economic output. When the environmental tax rate is low (e.g., $\tau_{p}\;$ = 0.05), the output per labor unit will rise resulting from the increase of the fraction of public health expenditure; however, when the environmental tax rate is high enough (e.g., $\tau_{p\;}$ = 0.25), the increase in the fraction of public health expenditure will decrease the output per labor unit.

### 5.3. Impact of the Combination of Policy Tools

**Figure 9:**
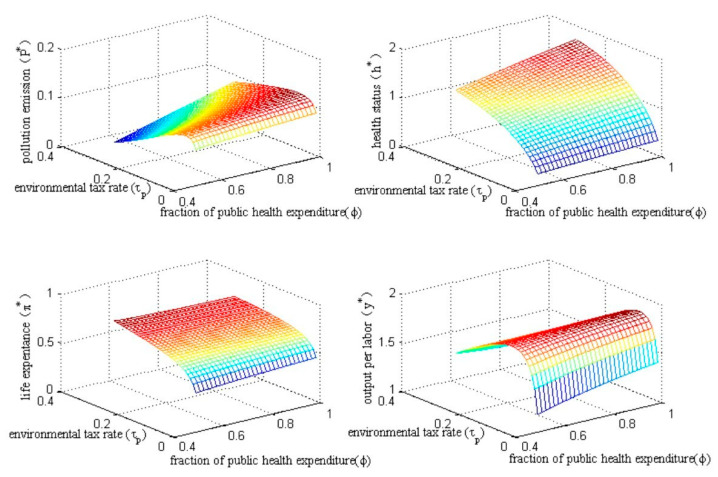
Impacts from the combination of $\tau_{p}$ and ϕ.

Environmental governance does not rely on a single policy tool. The government is more inclined to adopt a combination of multiple policy tools simultaneously to achieve multiple policy goals. In our model economy, the combination of environmental taxes and the fraction of public health spending may also produce different policy effects. In order to examine the impacts of the combination of environmental taxes and the fraction of public health expenditure, we simultaneously adjusted policy parameters $\tau_{p}$ and ϕ under the conditions of Proposition 1~Proposition 4. The results are shown in Figure 9.

From Figure 9 we can see different policy effects from the combination of environmental taxes and the fraction of public health expenditure on different endogenous variables. Specifically, the impact of the environmental tax rate on pollution emissions is inverted U-shaped under different fractions of public health expenditure, which once again shows that an increase in environmental tax rates will not necessarily result in a reduction in pollution emissions. The increased environmental tax rate will improve health status and life expectancy under different fractions of public health expenditure; however, the direction of the impact of the fraction of public health expenditure on health status and life expectancy will depend on the level of the environmental tax rate. In addition, the impact of the environmental tax rate on output per labor unit is also inverted U-shaped under different fractions of public health expenditure, which reconfirms the existence of the optimal environmental tax rate.

## 6. Conclusions

With the implementation of the Sustainable Development Goals in countries around the world, environmental governance has received more and more attention. Environmental governance policy objectives should not be limited to the elimination of polluting emissions. More importantly, governments should pay attention to the impact of environmental policies on health risks and economic output. In this paper, an overlapping generational general equilibrium model, including population structure change, was constructed to evaluate the impact of environmental governance on pollution emissions, health status, life expectancy, and economic output. The main findings of the article can be summed as follows: (i) The increase in pollution emissions per unit of output will not only lead to the deterioration of public health, but it will also hinder long-term economic growth, while the efficiency of pollution control will improve health and output per labor; (ii) Although levying environmental tax will improve health status and life expectancy, it has a non-linear impact on pollution emissions and output per labor unit, which means that there are trade-offs among environmental governance, public health improvements, and economic outputs; (iii) Although the increase in the proportion of public health expenditure will improve health status, its impact on life expectancy and economic output is affected by the level of environmental tax; only when the environmental tax rate is relatively low, will increasing the proportion of public health expenditure extend life expectancy and output per labor unit.

## Figures and Tables

**Table 1:** Calibration of parameters.

Parameters	Definition	Value
*A*	Total factor productivity	1.045^30^
α	Output elasticity of physical capital	0.60
β	Time-discount factor	0.99^30^
*b*	Adjustment parameter for life expectance	0.88
*n*	Number of children	0.65
*q*	Percentage of child-rearing cost on working income	0.10
*H*	Technology for the accumulation of health status	1.045^30^
μ	Depreciation rate for health status accumulation	0.85
σ	Output elasticity for health status accumulation	0.50
δ	Natural rate of pollution absorption	0.96
ρ	Degree of pollution induced by production	0.06
χ	Efficiency of pollution elimination	0.85
$\tau_{p}$	Environmental tax rate	0.046
ϕ	Fraction of public health expenditure	0.86

**Table 2:** Numerical results.

Variable	$\pi^{*}$	${\hat{s}}^{*}$	Annual Rate
Simulated value	0.5950	0.2857	4.45%
Real value (2020)	0.5976	0.2985	4.65%
Absolute errors	−0.0026	−0.0128	−0.20%
Relative errors	−0.44%	−4.29%	−4.30%

## Data Availability

Not applicable.

## Appendix A

**Table A1:** The environmental regulation laws in China after 2015.

Laws Related to Environmental Governance	Implementation Time or Last Revision Time
Environmental Protection Law of the People’s Republic of China	1 January 2015
Water Law of the People’s Republic of China	2 July 2016
Law of the People’s Republic of China on the Prevention and Control of Water Pollution	27 June 2017
Environmental Protection Tax Law of the People’s Republic of China	26 October 2018
Law of the People’s Republic of China on the Prevention and Control of Atmospheric Pollution	26 October 2018
Energy Conservation Law of the People’s Republic of China	26 October 2018
Environmental Impact Assessment Law of the People’s Republic of China	29 December 2018
Law of the People’s Republic of China on the Prevention and Control of Soil Pollution	1 January 2019
Law of the People’s Republic of China on the Prevention and Control of Solid Waste Pollution	29 April 2020
Law of the People’s Republic of China on the Prevention and Control of Solid Waste Pollution	1 September 2020
Law of the People’s Republic of China on the Prevention and Control of Noise Pollution	5 June 2022

Source: [71].
